# High systemic inflammation score is associated with adverse survival in skull base chordoma

**DOI:** 10.3389/fonc.2022.1046093

**Published:** 2022-10-14

**Authors:** Mingxuan Li, Jiwei Bai, Yujia Xiong, Yutao Shen, Shuai Wang, Chuzhong Li, Yazhuo Zhang

**Affiliations:** ^1^ Department of Neurosurgery, Beijing Tiantan Hospital, Capital Medical University, Beijing, China; ^2^ Beijing Neurosurgical Institute, Capital Medical University, Beijing, China; ^3^ Beijing Institute for Brain Disorders Brain Tumor Center, Beijing, China; ^4^ China National Clinical Research Center for Neurological Diseases, Beijing, China; ^5^ Key Laboratory of Central Nervous System Injury Research, Capital Medical University, Beijing, China

**Keywords:** systemic inflammation score, skull base chordoma, prognosis, biomarker, nomogram

## Abstract

**Background:**

The systemic inflammation score (SIS), based on preoperative lymphocyte to monocyte ratio (LMR) and albumin (ALB), was recently developed and is demonstrated to be a novel prognostic indicator in several cancers. However, data discussing the utility of SIS in chordoma are lacking. We aimed to investigate the distribution and the prognostic role of SIS in primary skull base chordoma patients undergoing surgery.

**Material and methods:**

Preoperative SIS was retrospectively collected from 183 skull base chordoma patients between 2008 and 2014 in a single center. Its associations with clinical features and overall survival (OS) were further analyzed. The SIS-based nomogram was developed and evaluated by the concordance index (C-index), time-dependent receiver operating characteristic (ROC) curve, calibration curve, and decision curve analysis (DCA).

**Results:**

The numbers of patients in the SIS 2, 1, and 0 group were 29 (15.8%), 60 (32.8%), 94 (51.4%), respectively. High SIS was associated with older age (*p* = 0.008), brainstem involvement of tumors (*p* = 0.039), and adverse OS (*p* < 0.001). Importantly, multivariate Cox analysis showed that high SIS independently predicts adverse OS. Furthermore, the nomogram based on SIS and clinical variables showed eligible performance for OS prediction in both training and validation cohorts.

**Conclusions:**

The SIS is a promising, simple prognostic biomarker, and the SIS-based nomogram serves as a potential risk stratification tool for outcome in skull base chordoma patients.

## Introduction

Chordoma accounts for 1-4% of primary bone neoplasm, and previous studies recognized that chordoma originates from the remnant of notochord (1, 2). Chordoma is largely found in the axial skeleton, about 30-40% of which is located at the skull base (clivus) (2, 3). To date, surgery with the help of radiotherapy is the main treatment for chordoma patients. However, the commonly large tumor burden and the involvement of critical neurovascular tissues make chordoma difficult to eradicate, especially for skull base chordoma (4). In addition, studies showed no significant value of classical chemotherapy. Despite the development of surgical technology, such as endoscopic surgery and intraoperative navigation, and the effort on targeted therapy, skull base chordoma patients had a high recurrent rate and sequent mortality (2, 5, 6). Improvement of the survival of chordoma patients requires the identification of reliable biomarkers and effective patient risk stratification.

Inflammation has been widely recognized as a prevalent characteristic of cancer since Virchow first promoted a potential association between inflammation and cancer (7). Cancer patients can present both local inflammatory symptoms and systemic inflammation, such as changes in the peripheral blood cell count (8). Moreover, increasing studies show that systemic inflammation plays an essential role in cancer patient outcomes *via* several aspects, including tumor oncogenesis, progression, and metastasis (9, 10). Consequently, several pretreatment inflammatory biomarkers based on blood cells, including neutrophil to lymphocyte ratio (NLR), prognostic nutritional index (PNI), lymphocyte to monocyte ratio (LMR), platelet to lymphocyte ratio (PLR), and Glasgow prognostic score (GPS), are developed in recent studies; and these biomarkers can act as prognostic indicators in various cancers (11–14). In addition, accumulating studies identify preoperative level of serum albumin (ALB) as a prognostic factor for cancer (15). However, there are currently few studies investigating the establishment of a scoring system that combines these inflammatory indicators for prognostic prediction in skull base chordoma patients (16).

More recently, a promising prognostic score based on levels of ALB and LMR, known as the systemic inflammation score (SIS), is proposed, and is highly associated with outcomes in colorectal cancer, oral cavity squamous cell carcinoma, and clear cell renal cell carcinoma (17–19). Until now, the role of SIS in predicting the prognosis of skull base chordoma patients remains to be elucidated. Thus, in this study, we evaluated the distribution of SIS in a relatively large cohort of skull base chordoma patients undergoing surgery and investigated the associations of SIS with patient characteristics. We also examined the prognostic impact of SIS on overall survival (OS) in skull base chordoma patients, and a nomogram including SIS and clinical variables was further developed and validated for survival prediction.

## Material and methods

### Patient population

This retrospective study enrolled 183 skull base chordoma patients undergoing surgery between January 2008 and September 2014 in our institute. This cohort has been previously described in our studies (20). The following inclusion criteria were applied: 1) patients were histopathologically diagnosed as chordoma and the tumor located at the skull base. 2) patients with detailed medical records, routine blood tests before surgery, and follow-up data. Exclusion criteria were as follows: 1) patients with an unclear diagnosis or tumors located at the cervical vertebra alone were not included; 2) patients who received adjuvant radiotherapy and/or chemotherapy, cancer-related operation including biopsy before their first surgery at our institute were excluded; 3) unavailable clinical/laboratory data; 4) patients with infection, inflammatory disease, hemopathy, liver/kidney disorder, other malignant diseases were excluded.

### Follow-up investigation and survival analysis

Patients were recommended to follow up with physical examination, magnetic resonance imaging, and/or computed tomography every 3-6 months for the first 2 years and annually thereafter. For patients unable to come to our hospital for a check, telephone, or email was applied. The follow-up investigation was terminated in October 2019. The primary endpoint, OS, was defined as the time from operation to death or censored at the last follow-up.

For survival analysis and the development of the nomogram, 183 patients were divided into training and validation groups with a ratio of 2:1.

### Data extraction

For each participant, clinical and pathological data and blood tests were gathered, including age at surgery, sex, pathological type, degree of resection, tumor size, tumor texture, tumor blood supply, brainstem involvement, preoperative complete blood cell count, and ALB level (g/L). Pathological types of chordoma were confirmed by a professional pathologist and recorded as classical, chondroid, or dedifferentiated types (2). The degree of resection was recorded as total/subtotal resection (≤ 5% residual tumor) and partial resection (> 5% residual tumor) according to the postoperative images (21).

### Definition of SIS

Given the contiguous features of preoperative LMR and ALB, the optimal cutoff values, determined by the “surv_cutpoint” function of R package “survminer”, were used to transfer LMR and ALB into categorical variables (high level, > the cutoff value; and low level, ≤ the cutoff value). The threshold point was defined as the value with the maximally selected log-rank test statistics (22). The SIS was then defined based on ALB and LMR levels as previously described: score 0, patients with a high LMR and a high ALB; score 1, patients with either low LMR or low ALB; and score 2, patients with both low ALB and low LMR (17, 19).

### Statistical analysis

The analysis was performed with SPSS for Windows version 19.0 (IBM Corp, Armonk, NY, USA), and R software version 4.0.2 (R Foundation for Statistical Computing, Vienna, Austria). Continuous variables were shown as median with interquartile range (IQR). Correlations between SIS, LMR, and categorical variables were analyzed by the chi-squared test, and their correlations to continuous variables were analyzed using the Kruskal-Wallis test or Wilcoxon rank-sum test. The Kaplan-Meier survival curve and log-rank test were applied to find the difference in OS between groups. Univariate and multivariate Cox proportional hazard regression model was also performed, and variables with *P* < 0.05 in the univariate analysis were included in the multivariate analysis. A nomogram was developed based on our multivariate analysis and previously reported prognostic-related variables (2, 4, 23). The concordance index (C-index), time-dependent receiver operating characteristic (ROC) curve, and the calibration curve were used to assess the predictive performance and accuracy of the nomogram. Decision curve analysis (DCA) was performed to evaluate the clinical value of the nomogram. A two-sided *P* value less than 0.05 was considered to indicate statistical significance.

## Results

### Patient characteristics

In total, 183 patients were included in this study. **Supplementary Table S1** showed the clinicopathological characteristics of enrolled patients. There were 96 (52.5%) males and 87 (47.5%) females. The median age at admission was 41 years (IQR, 29-51 years). Respectively, 125 (68.3%), 58 (31.7%), 0 (0%) patients had classical, chondroid, and dedifferentiated chordoma. Based on the longest diameters of the coronal, sagittal, and axial axes, the median tumor size was 21.0 cm^3^ (IQR, 11.9-38.8 cm^3^). The median value of preoperative LMR was 5.35 (IQR, 4.17-6.75), and the median level of preoperative ALB was 45.8 g/L (IQR, 43.9-48.2 g/L). The excellent cutoff value of LMR was 4.75, and 44.5 g/L for ALB in the training cohort (**Supplementary Figure S1**). According to the above definition of SIS, the distribution of SIS in our cohort was 29 (15.8%) patients in the SIS 2 group, 60 (32.8%) patients with an SIS of 1, and 94 (51.4%) patients with an SIS of 0.

### Correlations between SIS and clinicopathological findings

Relationships between SIS and clinicopathological variables were detailed in **Table 1**. Higher SIS was significantly correlated with older patient age (*p* = 0.008). In addition, patient with brainstem involvement was associated with a higher SIS (*p* = 0.039). Patients with higher SIS tended to have tumors with a rich blood supply, although the difference was not significant (*p* = 0.096). No significant differences in gender, tumor size, tumor texture, and pathology types were observed between different SIS groups (all *p* > 0.05). Additionally, we observed that low ALB was associated with older age (*p <*0.001), though no significant difference between low LMR and old age was found (*p* = 0.316) (20). Decreased ALB was also correlated with soft tumors and chondroid chordoma types (*p* = 0.023 and 0.026, respectively) (20), while LMR was not associated with tumor texture (*p* = 0.094) or pathological type (*p* = 0.311).

**Table 1:** Association between SIS and clinicopathological features in skull base chordoma.

Variables	SIS			
	0	1	2	*P* value
Cases	94	60	29	
Age, years				0.008
Median	37.0	43.5	47.0	
Gender				0.889
Male	48	33	15	
Female	46	27	14	
Tumor size				0.814
≤20cm^3^	45	31	13	
>20cm^3^	49	29	16	
Texture				0.992
Soft	28	18	9	
Others (hard or moderate)	66	42	20	
Blood supply				0.096
Rich	50	35	22	
Others (poor or moderate)	44	25	7	
Pathology				0.634
Classical	63	40	22	
Chondroid	31	20	7	
Brainstem involvement				0.039
Absent	30	30	8	
Present	64	30	21	

SIS, systemic inflammation score.

### Survival analysis

**Figure 1:**
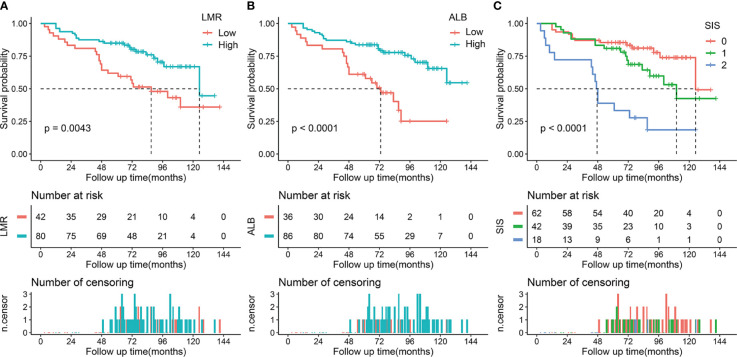
Kaplan-Meier survival analysis of LMR, ALB, and SIS in the training cohort. **(A)** Low LMR correlated with a poor OS. **(B)** Low ALB correlated with an adverse OS. **(C)** High SIS was associated with a poor OS. LMR, lymphocyte to monocyte ratio; ALB, albumin; SIS, systemic inflammation score; OS, overall survival.

**Figure 2:**
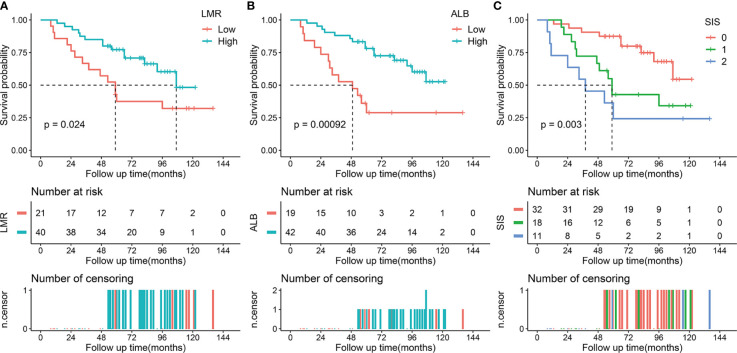
Kaplan-Meier survival analysis of LMR, ALB, and SIS in the validation cohort. **(A)** Low LMR correlated with a poor OS. **(B)** Low ALB correlated with an adverse OS. **(C)** High SIS was associated with a poor OS. LMR, lymphocyte to monocyte ratio; ALB, albumin; SIS, systemic inflammation score; OS, overall survival.

The median follow-up period was 74 months (IQR, 53-96 months; range, 3-141 months). During the follow-up period, 72 (39.3%) patients died and the 5-year OS rate was 67.8% for the whole cohort. In the training cohort, Kaplan-Meier curves indicated that decreased LMR was associated with shorter OS time (median OS time, 87 months vs 125 months; 5-year OS rate, 61.9% vs 85.0%; *p* = 0.004) (**Figure 1A**). Similarly, patients with low ALB had adverse OS than that of patients with high ALB (median OS time, 73 months vs not reached; 5-year OS rate, 61.1% vs 83.7%; *p* < 0.001, **Figure 1B**) (20). Moreover, we found that patients with higher SIS were correlated with worse OS (*p* < 0.001, **Figure 1C**). Specifically, significant differences of OS were observed between SIS = 2 group and SIS = 1 group (median OS time, 47 months vs 110 months; 5-year OS rate, 38.9% vs 81.0%; *p* = 0.001) or SIS = 0 group (median OS time, 47 months vs 125 months; 5-year OS rate, 38.9% vs 85.5%; *p* < 0.001). In the validation cohort, we also observed that low LMR was correlated with shorter OS time (median OS time, 59 months vs 107 months; 5-year OS rate, 52.4% vs 77.2%; *p* = 0.024) (**Figure 2A**). Additionally, low ALB was associated with decreased 5-year OS rate (median OS time, 49 months vs not reached; 5-year OS rate, 36.1% vs 78.1%; *p* = 0.001) (**Figure 2B**). Importantly, patients with higher SIS were correlated with worse OS outcome (median OS time, 38 months, 59 months, and not reached, respectively; 5-year OS rate, 87.5%, 42.8%, and 24.2%, respectively; *p* = 0.003) (**Figure 2C**).

**Figure 3:**
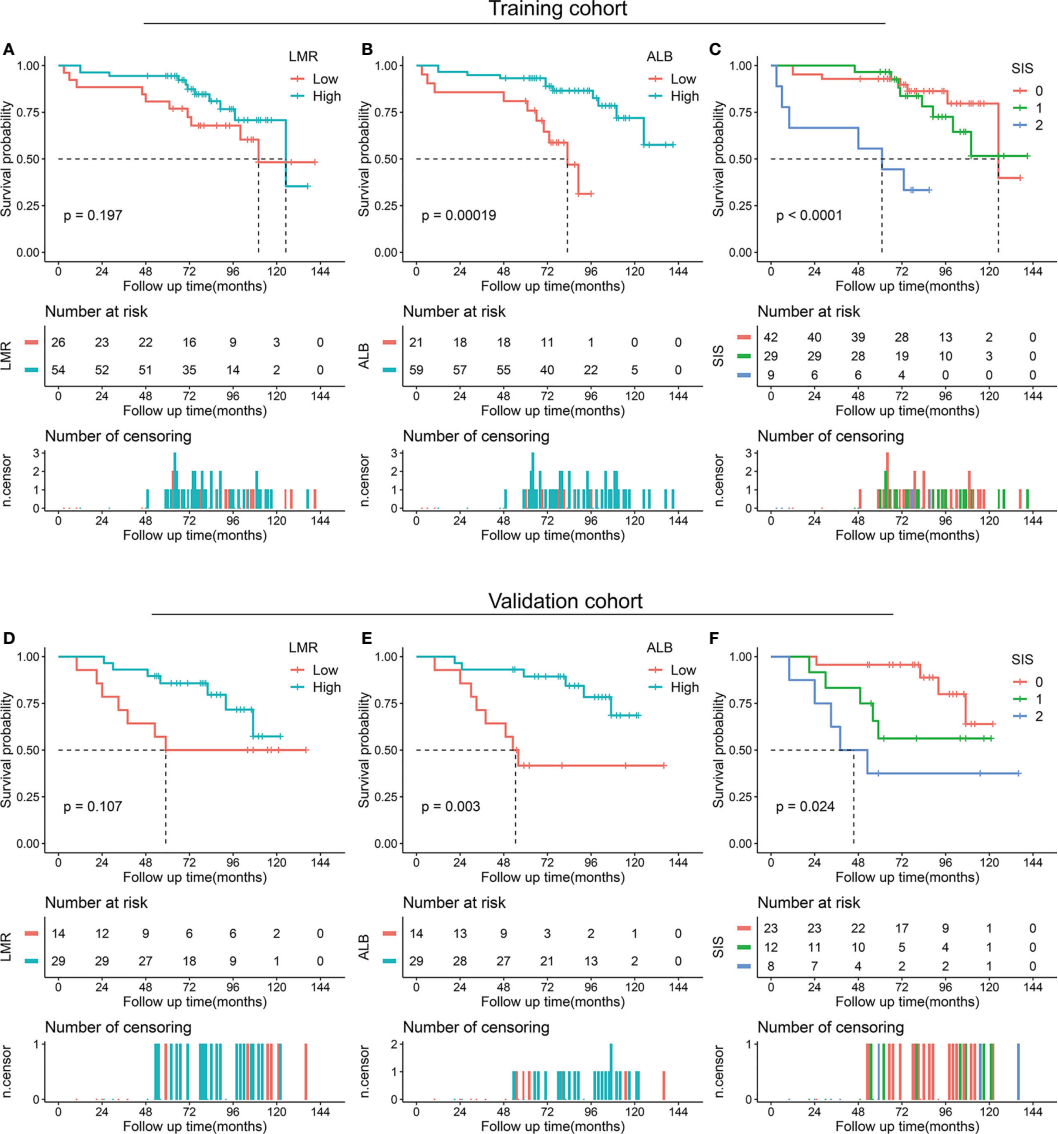
Kaplan-Meier survival analysis of LMR, ALB, and SIS in skull base chordoma with total or subtotal resection. **(A)** OS analysis of LMR in the training cohort. **(B)** OS analysis of ALB in the training cohort. **(C)** OS analysis of SIS in the training cohort. **(D)** OS analysis of LMR in the validation cohort. **(E)** OS analysis of ALB in the validation cohort. **(F)** OS analysis of SIS in the validation cohort. LMR, lymphocyte to monocyte ratio; ALB, albumin; SIS, systemic inflammation score; OS, overall survival.

**Figure 4:**
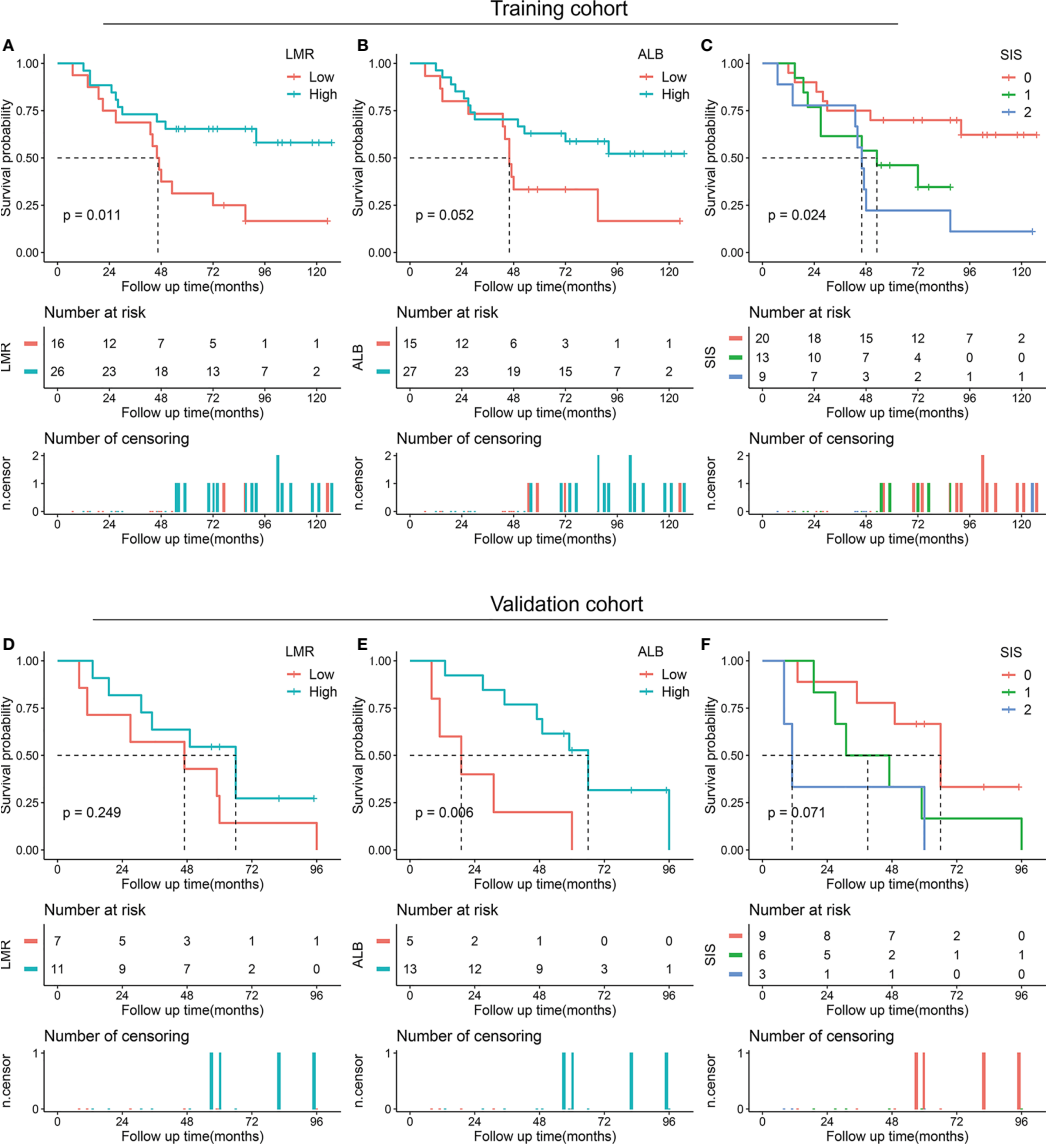
Kaplan-Meier survival analysis of LMR, ALB, and SIS in skull base chordoma with partial resection. **(A)** OS analysis of LMR in the training cohort. **(B)** OS analysis of ALB in the training cohort. **(C)** OS analysis of SIS in the training cohort. **(D)** OS analysis of LMR in the validation cohort. **(E)** OS analysis of ALB in the validation cohort. **(F)** OS analysis of SIS in the validation cohort. LMR, lymphocyte to monocyte ratio; ALB, albumin; SIS, systemic inflammation score; OS, overall survival.

We also performed a subgroup analysis of LMR, ALB, and SIS stratified by degree of resection. For patients with total/subtotal resection, decreased ALB (*p* < 0.001 in the training cohort and *p* = 0.003 in the validation cohort) and high SIS (*p* < 0.001 in the training cohort and *p* = 0.024 in the validation cohort) were associated with unfavorable OS, low LMR tended to correlate with poor OS (*p* = 0.197 in the training cohort and *p* = 0.107 in the validation cohort) (**Figure 3**). For patients with partial resection, we also confirmed the potential prognostic value of LMR (*p* = 0.011 in the training cohort), ALB (*p* = 0.052 in the training cohort and *p* = 0.006 in the validation cohort), and SIS (*p* = 0.024 in the training cohort and *p* = 0.071 in the validation cohort) (**Figure 4**).

In the univariate Cox analysis of the training cohort, LMR, ALB, and SIS were associated with OS as well as age at admission, tumor pathology, degree of resection, and lymphocyte count. Moreover, multivariate analysis including these variables revealed that SIS was an independent prognostic indicator for OS (**Table 2**). Consistently, in the validation cohort, higher SIS was independently associated with adverse survival (**Table 3**).

**Table 2:** Univariate and multivariate Cox proportional analysis of OS in the training cohort.

Variables	Univariate analysis		Multivariate analysis	
	HR (95% CI)	*P* value	HR (95% CI)	*P* value
Age, years (>55 vs ≤55)	2.626 (1.345-5.128)	0.005	2.895 (1.412-5.935)	0.004
Gender (female vs male)	0.904 (0.500-1.634)	0.738		
Tumor size, cm^3^(>20 vs ≤20)	1.593 (0.873-2.906)	0.129		
Texture (hard + moderate vs soft)	1.154 (0.603-2.207)	0.666		
Blood supply (poor + moderate vs rich)	0.536 (0.281-1.026)	0.060		
Pathology (chondroid vs classical)	0.303 (0.128-0.717)	0.007	0.385 (0.159-0.913)	0.034
Brainstem involvement (present vs absent)	0.938 (0.512-1.717)	0.835		
Degree of resection (partial vs total/subtotal)	2.696 (1.488-4.883)	<0.001	2.085 (1.123-3.870)	0.020
Postoperative radiotherapy (yes vs no)	0.880 (0.482-1.607)	0.678		
Neutrophil count (10^9^/L) ^a^	0.996 (0.798-1.242)	0.969		
Lymphocyte count (10^9^/L) ^a^	0.521 (0.305-0.891)	0.017	NA	
Monocyte count (10^9^/L) ^a^	0.244 (0.028-2.115)	0.200		
Platelet count (10^9^/L) ^a^	0.999 (0.994-1.004)	0.586		
LMR (≤4.75 vs >4.75)	2.309 (1.277-4.184)	0.006	NA	
ALB (≤44.5 vs >44.5)	3.370 (1.827-6.216)	<0.001	NA	
SIS				
0	Reference		Reference	
1	1.827 (0.891-3.749)	0.100	1.702 (0.799-3.626)	0.168
2	5.848 (2.756-12.406)	<0.001	4.626 (2.124-10.074)	<0.001

a analyzed as continuous variables; NA, not acquired; OS, overall survival; HR, hazard ratio; CI, confidence interval; LMR, lymphocyte to monocyte ratio; ALB, albumin; SIS, systemic inflammation score.

**Table 3:** Univariate and multivariate Cox proportional analysis of OS in the validation cohort.

Variables	Univariate analysis		Multivariate analysis	
	HR (95% CI)	*P* value	HR (95% CI)	*P* value
Age, years (>55 vs ≤55)	1.169 (0.443-3.085)	0.752		
Gender (female vs male)	1.204 (0.564-2.568)	0.632		
Tumor size, cm^3^(>20 vs ≤20)	1.728 (0.793-3.765)	0.168		
Texture (hard + moderate vs soft)	2.711 (0.936-7.850)	0.066		
Blood supply (poor + moderate vs rich)	0.510 (0.223-1.167)	0.111		
Pathology (chondroid vs classical)	0.932 (0.430-2.019)	0.857		
Brainstem involvement (present vs absent)	1.245 (0.573-2.706)	0.580		
Degree of resection (partial vs total/subtotal)	3.907 (1.812-8.423)	0.001	4.193 (1.934-9.089)	<0.001
Postoperative radiotherapy (yes vs no)	0.581 (0.246-1.370)	0.215		
Neutrophil count (10^9^/L) ^a^	1.434 (1.146-1.793)	0.002	NA	
Lymphocyte count (10^9^/L) ^a^	1.253 (0.681-2.302)	0.468		
Monocyte count (10^9^/L) ^a^	3.357 (0.325-34.622)	0.309		
Platelet count (10^9^/L) ^a^	1.005 (1.001-1.010)	0.018	NA	
LMR (≤4.75 vs >4.75)	2.306 (1.093-4.865)	0.028	NA	
ALB (≤44.5 vs >44.5)	3.364 (1.573-7.195)	0.002	NA	
SIS				
0	Reference		Reference	
1	2.807 (1.158-6.808)	0.022	2.697 (1.108-6.564)	0.029
2	4.480 (1.712-11.720)	0.002	5.167 (1.962-13.610)	0.001

a analyzed as continuous variables; NA, not acquired; OS, overall survival; HR, hazard ratio; CI, confidence interval; LMR, lymphocyte to monocyte ratio; ALB, albumin; SIS, systemic inflammation score.

### Prognostic nomogram for OS

**Figure 5:**
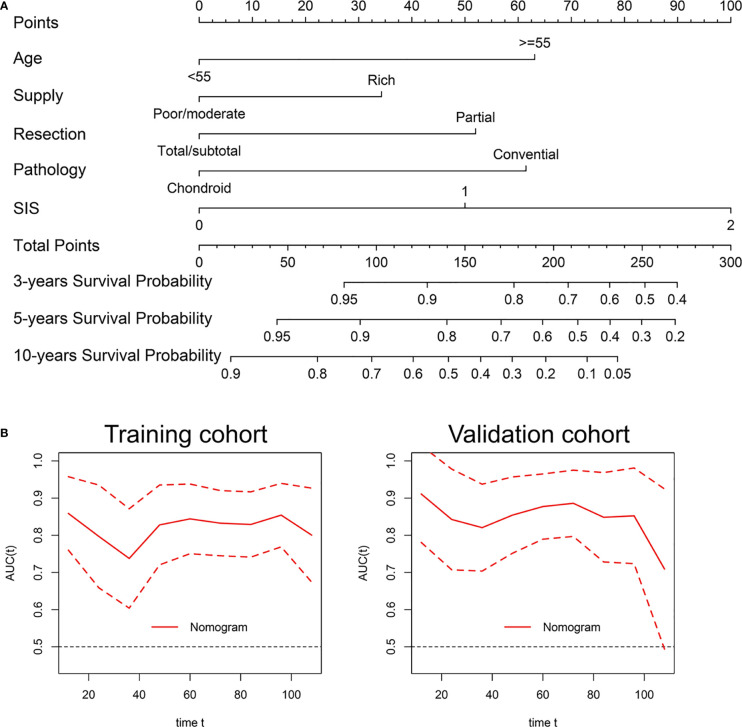
Development and validation of a nomogram for OS prediction in skull base chordoma. **(A)** Nomogram for OS prediction. **(B)** Time-dependent ROC curve of the nomogram. SIS, systemic inflammation score; OS, overall survival; ROC, receiver operating characteristic.

**Figure 6:**
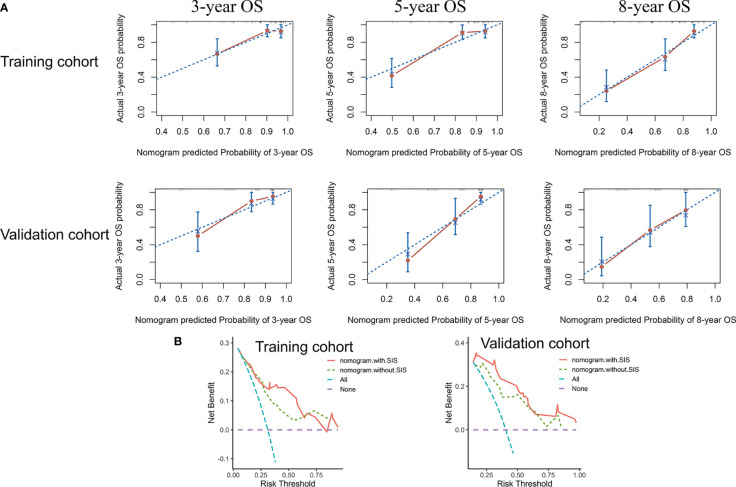
The nomogram showed satisfactory performance for OS prediction in the training and validation cohort. **(A)** 3-year, 5-year, and 8-year OS calibration curves of the nomogram. **(B)** Decision curve analysis of the nomogram for OS. OS, overall survival.

We then developed the nomogram integrating SIS and significant clinical variables for clinical OS prediction (**Figure 5A**). The C-indexes of the nomogram were 0.791 (95% CI, 0.720-0.861) in the training cohort and 0.798 (95% CI, 0.727-0.868) in validation cohort. Time-dependent ROC also revealed the satisfying prediction performance of the SIS-based nomogram during the follow-up time (**Figure 5B**). Moreover, calibration curves for 3-year, 5-year, and 8-year OS suggested favorable agreements between the nomogram and actual observation in both training and validation cohorts (**Figure 6A**). Importantly, DCA, a tool to assess the clinical values of models, showed that the SIS-based nomogram had higher net benefits (**Figure 6B**).

## Discussion

In the current study, we described the distribution of SIS in newly diagnosed skull base chordoma and investigated its associations with the clinical features of patients. To our knowledge, the current study was the first report to characterize the prognostic role of SIS in chordoma. Our results showed that higher SIS was correlated with older patient age and brainstem involvement of the tumor, and the multivariate analysis demonstrated that high SIS could independently predict adverse OS. Moreover, a nomogram including SIS and significant clinical variables showed a favorable prediction ability for OS. Our data highlighted the SIS-based nomogram may be of important value for clinical survival prediction, and further supported that drugs targeting inflammation may be a promising therapy for chordoma.

The role of inflammation, a prevalent characteristic of cancer, on tumor pathogenesis and progression has gained much attention in recent years (7, 24). Increasing research has indicated that inflammatory cytokines in tumors, including tumor-infiltrating lymphocyte and tumor-infiltrating neutrophils, play essential roles in tumor oncogenesis and patient outcome (25, 26). Moreover, tumor-associated macrophage was also correlated with poorer outcome and therapy resistance in cancer (27). In addition, the peripheral blood cells of cancer patients, such as neutrophil, monocyte, and lymphocyte, and further pretreatment NLR, PLR, and LMR showed effective prognostic values in various cancers (11, 13). Recently, one retrospective study by Hu et al. investigated the role of inflammatory indexes including NLR, PLR, and monocyte lymphocyte ratio (MLR) in 172 chordoma patients (16). However, the authors found that preoperative MLR was not correlated with patient OS, which is inconsistent with the finding in this study. Our result of Kaplan-Meier analyses showed that low LMR was associated with unfavorable OS in skull base chordoma (*p* = 0.004 in the training cohort and 0.024 in the validation cohort). Several differences exist in the current study compared to Hu’s study, which may help explain the inconsistency. First, the study populations differed from each other; in the study of Hu, 79 skull base chordoma, 43 spine chordoma, and 50 sacrum chordoma were enrolled while our study only involved skull base chordoma. Spine chordoma often correlated with metastasis while limited metastasis was reported in skull base chordoma patients, and metastasis was identified as a significant prognostic factor for outcome (2, 28). Given the potential differences between spine chordoma and skull base chordoma, the results may be biased by the analysis of the entire cohort without stratification by tumor location. Second, the cut-off values were not consistent; the cutoff value of MLR was 0.36 (LMR, 2.78) in the study of Hu, while the cutoff value of LMR in the current study was 4.75. Finally, methods determining cutoff values were not the same; R package “survminer” was used in the current study while ROC analysis was applied in Hu’s study.

SIS attracts accumulating attention in recent studies for its integration of ALB and LMR, which may indicate the status of both nutrition and systemic inflammation in patients (17). Increasing studies have shown the prognostic value of SIS in cancer patients (19, 29). In our study, similar to previous studies, we found high SIS was correlated with older age and brainstem invasion, and SIS was independently associated with OS, identifying SIS as a novel risk stratification system for skull base chordoma. Given the difficulty and high risk of surgery involving the brainstem, patients with a high SIS may need a more careful preoperative preparation such as navigation-assisted operation, and electrophysiological monitoring, aiming for maximal safe resection and less possibility of recurrence. Moreover, patients with high SIS are recommended to receive closer monitoring after surgery and a more radical therapeutic option due to the increased risk of death (SIS = 1 vs SIS = 0, 1.702 times; SIS = 2 vs SIS = 0, 4.626 times in the training cohort).

The reason for the prognostic role of SIS in cancer is largely unclear, and previous studies proposed that it may be elucidated by the role of lymphocyte, monocyte, and ALB. The lymphocyte is recognized as an essential component of the immune system and it contributes to immune surveillance of cancer cells, inhibition of tumor cell growth, and metastasis *via* secreting various cytokines (30). A decrease in lymphocytes may lead to an insufficiency of the immune response to cancer, and it is correlated with poor outcomes in various cancers (31). In contrast, monocyte-derived tumor-associated macrophage in tumor tissues can act as a cancer-promoting role by enhancing tumor cell proliferation, increased angiogenesis, stroma remodeling, and inhibiting antitumor immunity (32). Moreover, monocyte count is correlated with survival in cancer patients (33). Therefore, the association between low LMR and adverse outcome is found in cancer patients in recent studies (13). ALB, which is regarded as an index of nutrition, is also a negative acute phase protein involved in systemic inflammation response (10). Previous studies indicated the association between low ALB and poor prognosis, which may be explained by the abnormal response to surgical stress, insufficient immune defense, and increased risks of complications (34).

Several limitations existed in this study. First, retrospective nature may be subject to selection bias. The prognostic role of SIS in chordoma remains to be elucidated in future large-scale research. In addition, preoperative C-reactive protein and pretreatment GPS were not collected due to the not routine measurement in examinations; and their associations with SIS were not analyzed in this study, while the previous study showed the prognostic value of C-reactive protein in chordoma (35). Finally, the associations between SIS and inflammatory cells in tumor tissues such as tumor-infiltrating lymphocyte were not analyzed (36).

In summary, our results reveal that SIS is an independent prognostic indicator of OS in skull base chordoma, and the SIS-based nomogram can act as a clinically preoperative risk stratification tool for the decision-making of individualized therapy for skull base chordoma patients.

## Data availability statement

The original contributions presented in the study are included in the article/**Supplementary Material**. Further inquiries can be directed to the corresponding author.

## Ethics statement

The studies involving human participants were reviewed and approved by the Ethics Committee of Beijing Tiantan Hospital. The patients/participants provided their written informed consent to participate in this study.

## Author contributions

ML, JB, and YZ contributed to the conception and design of the study. YX, YS, SW, and CL contributed to data collection, analysis and interpretation of data, and revision of the manuscript. ML wrote the manuscript. All authors read and approved the final manuscript.

## Funding

This study was supported by the National Natural Science Foundation of China (82071559 and 82272939).

## Acknowledgments

We are grateful for the support of all patients.

## Conflict of interest

The authors declare that the research was conducted in the absence of any commercial or financial relationships that could be construed as a potential conflict of interest.

## Publisher’s note

All claims expressed in this article are solely those of the authors and do not necessarily represent those of their affiliated organizations, or those of the publisher, the editors and the reviewers. Any product that may be evaluated in this article, or claim that may be made by its manufacturer, is not guaranteed or endorsed by the publisher.

## Abbreviations

LMR, lymphocyte to monocyte ratio; ALB, albumin; SIS, systemic inflammation score; OS, overall survival; HR, hazard ratio; CI, confidence interval; C-index, concordance index; ROC, receiver operating characteristic; DCA, decision curve analysis.
