# Supplementary figures and images for: High systemic inflammation score is associated with adverse survival in skull base chordoma
Source: Front Oncol. 2022 Oct 14;12:1046093. doi: 10.3389/fonc.2022.1046093 (PMC9613931; doi:10.3389/fonc.2022.1046093)

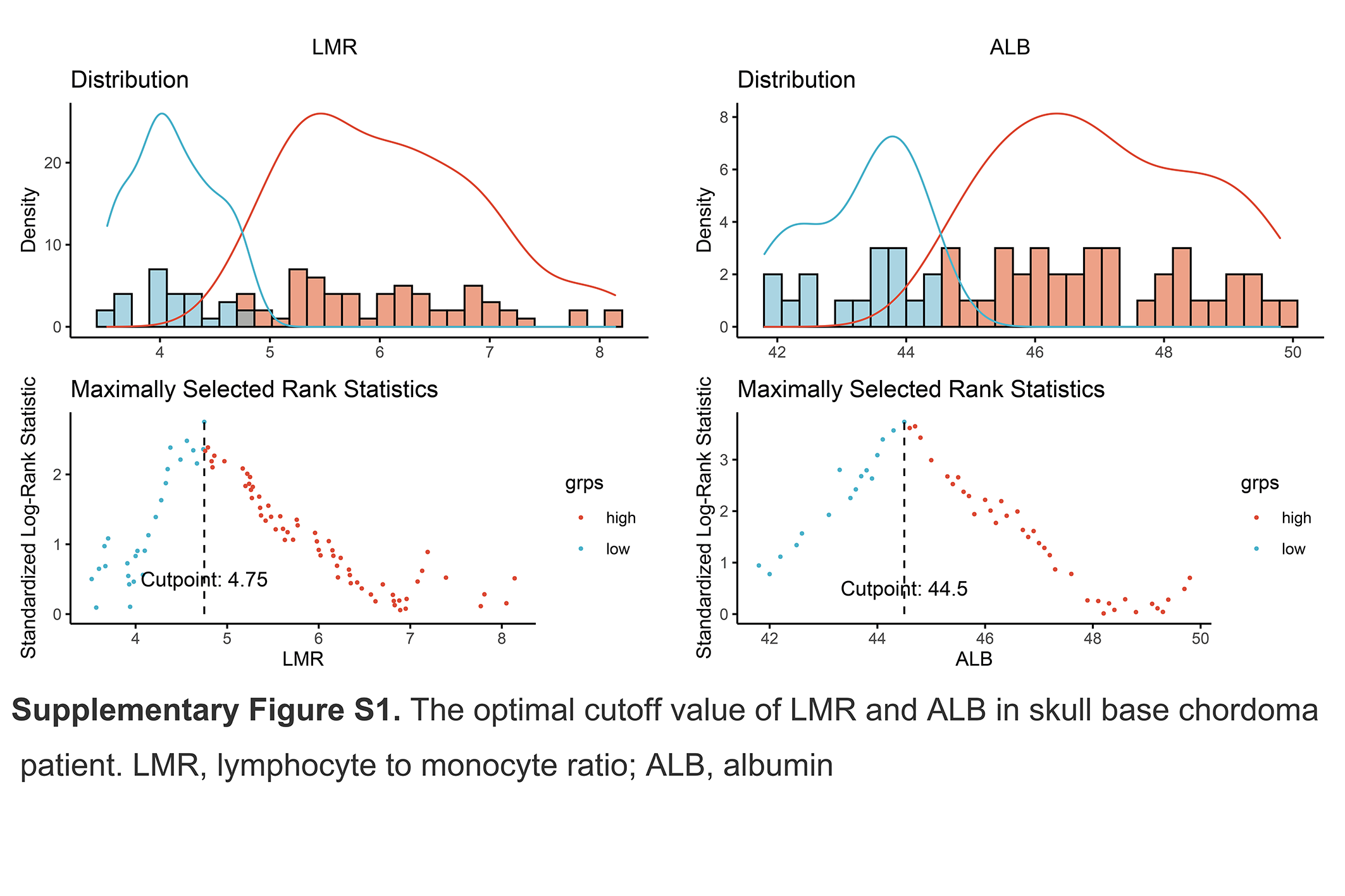

Supplement: Supplementary file 1 [file Image_1.tif]
