# Supplementary material for: High systemic inflammation score is associated with adverse survival in skull base chordoma
Source: Front Oncol. 2022 Oct 14;12:1046093. doi: 10.3389/fonc.2022.1046093 (PMC9613931; doi:10.3389/fonc.2022.1046093)
Supplement: Supplementary file 2 [file Table_1.docx]

**Table S1** Summary of clinicopathological characteristics of 183 enrolled skull base chordoma patients

| Characteristic | Number of patients (%) |
| --- | --- |
| Median age (year, IQR) | 41 (29-51) |
| Sex |  |
| Male | 96 (52.5) |
| Female | 87 (47.5) |
| Median tumor size (cm^3^, IQR) | 21.0 (11.9-38.8) |
| Tumor texture |  |
| Soft | 55 (30.1) |
| Others (hard or moderate) | 128 (69.9) |
| Tumor blood supply |  |
| Rich | 107 (58.5) |
| Others (poor or moderate) | 76 (41.5) |
| Pathology type |  |
| Classical | 125 (68.3) |
| Chondroid | 58 (31.7) |
| Dedifferentiated | 0 (0) |
| Brainstem involvement |  |
| No | 68 (37.2) |
| Yes | 115 (62.8) |
| Median neutrophil count (10^9^/L, IQR) | 3.47 (2.67-4.46) |
| Median lymphocyte count (10^9^/L, IQR) | 1.86 (1.54-2.30) |
| Median monocyte count (10^9^/L, IQR) | 0.35 (0.28-0.45) |
| Median platelet count (10^9^/L, IQR) | 234 (197-265) |
| Median ALB (g/L, IQR) | 45.8 (43.9-48.2) |
| Median LMR (IQR) | 5.35 (4.17-6.75) |
| Recurrence during follow up | 128 (69.9) |
| Death during follow up | 72 (39.3) |
| Median follow up time (month, IQR) | 74 (53-96) |

IQR, interquartile range; LMR, lymphocyte to monocyte ratio; ALB, albumin
